# Pathology of incipient neoplasia (3rd edition)

**DOI:** 10.1038/sj.bjc.6600170

**Published:** 2002-03-18

**Authors:** C Fisher

**Affiliations:** The Royal Marsden Hospital, Fulham Road, London SW3 6JJ, UK

## Abstract

*British Journal of Cancer* (2002) **86**, 1019. DOI: 10.1038/sj/bjc/6600170 www.bjcancer.com

© 2002 Cancer Research UK

## 

**Edited by DE Henson and J Albores-Saavedra. Publishers: Oxford University Press, 2001. ISBN 0-19-512338-7. £120. 839 pages.**

Some cancers can be prevented if their precursor lesions are detected and eradicated, or cured by removal at an early stage. Pathologists therefore spend an increasing proportion of their time on the diagnosis and grading of early cancer or of premalignant or preinvasive lesions. There are also benign neoplasms in which malignant change can develop and, in a wider context some, such as soft tissue sarcomas, in which progression of malignant grade can occur.

The aim of this book, now in its third edition, is to describe incipient lesions, and to provide morphologic criteria for their diagnosis and progression as well as clues useful in the differential diagnosis. Because light microscopy remains the basis for diagnosis, the authors define incipient as referring to well defined histologic changes that have an increased probability or risk for cancer. These include hyperplasia, dysplasia, metaplasia carcinoma *in situ* and also minimal or even microinvasive carcinoma. Most of the authors are recognised experts in their field, but since the book is arranged by organ system, their brief is interpreted and applied in different ways. Those chapters concerned with epithelial lesions are most clearly related to the stated purpose of the book. The sections on gastrointestinal tumours (especially those on small and large intestine which include guidelines for reporting), lung tumours, skin, endometrium and cervix are well presented and useful. The chapter on breast includes a detailed and substantial discussion of ductal carcinoma *in situ*, principally from the point of view of the author's classification. In other areas, such as mesenchymal and neural tissue, concepts of incipient neoplasia have (as the authors acknowledge) not been defined and the histological precursors of malignant tumours are not clearly defined. For some of these, therefore, the respective authors have either focused on descriptions of benign or low grade neoplasms, or emphasised aspects of molecular carcinogenesis. Thus, in the chapter on bone tumours, the authors consider pre-existing conditions and aetiologic factors together and the account of soft tissue tumours concerns benign to malignant transformations, progression from low-grade to high-grade malignancies, and specific chromosomal translocations. While these chapters are informative, it is difficult to regard synovial sarcoma and radiation-induced sarcoma, for example, as incipient. In some chapters genetic findings have been neatly integrated with morphologic description, and in others they are given more prominence: there is a 10 page account of the molecular genetics of bladder cancer, including a complete map of chromosome 9. There are some quirks of content and organisation. A section on the eye is included, but among the gynaecological chapters there is no account of vulval or vaginal lesions. Unaccountably, a whole chapter is devoted to neuroblastoma including screening. Wilms tumour has a separate chapter from other neoplasms of the kidney. As might be expected, there are abundant illustrations, with a mixture of colour and black and white micrographs, mostly well chosen and reproduced, although some of the background colours are rather heterogeneous. The print, however, is slightly reader-unfriendly, with a small serif font on shiny paper.

Although the book is not exhaustive nor a reference work, it generally achieves its purpose and indeed goes beyond it. To some extent, this imparts a mixed quality. For the diagnostic pathologist, it does not really serve as a practical bench book except for selected areas; and from the standpoint of a scientist, it is primarily a morphologic text that presupposes training in diagnostic histopathology. In a future edition, therefore, the aim needs to be broadened and the title perhaps amended to reflect this. Nevertheless, this edition is packed with information (at 2.25 kg it is unexpectedly heavy for its size) and reasonably up to date and in addition to its relevance for pathologists would be useful reading for oncologists, other clinicians, and scientists dealing with or researching into cancer.

